# Next-Generation Sequencing of *Mycobacterium tuberculosis*

**DOI:** 10.3201/eid2206.152051

**Published:** 2016-06

**Authors:** Igor Mokrousov, Ekaterina Chernyaeva, Anna Vyazovaya, Viacheslav Sinkov, Viacheslav Zhuravlev, Olga Narvskaya

**Affiliations:** St. Petersburg Pasteur Institute, St. Petersburg, Russia (I. Mokrousov, A. Vyazovaya, O. Narvskaya);; St. Petersburg State University, St. Petersburg (E. Chernyaeva);; Research Institute of Phthisiopulmonology, St. Petersburg (E. Chernyaeva, V. Zhuravlev, O. Narvskaya);; Scientific Center of Family Health and Reproductive Problems, Irkutsk, Russia (V. Sinkov

**Keywords:** whole-genome sequencing, next-generation sequencing, Mycobacterium tuberculosis, spoligotyping, bacteria, tuberculosis and other mycobacteria

**To the Editor:** Next-generation sequencing (NGS) technology is becoming more affordable and is increasingly being widely used for high-resolution molecular epidemiology of tuberculosis. Using an example of the emerging multidrug-resistant strain of *Mycobacterium tuberculosis*, we showed the value of informed understanding when in silico prediction from NGS data achieved with available bioinformatics tools is placed within the context of the existing genotyping framework.

Spoligotyping is a classical method of *M. tuberculosis* genotyping, and the SITVIT_WEB database contains data on 7,105 spoligotype patterns of 58,180 isolates from 153 countries (http://www.pasteur-guadeloupe.fr:8081/SITVIT_ONLINE). Spoligotyping targets a variation of the DR/CRISPR locus, whose evolution in *M. tuberculosis* occurs through deletion of single or multiple spacers. By virtue of the orientation of the associated *cas* genes, the locus is situated on the minus strand, whereas its spacers are numbered within the locus, not the genome. Spoligotype international type (SIT) 266 (Figure, panel A) is an epidemiologically significant genotype. It constitutes a substantial proportion of the population structure of *M. tuberculosis* in Belarus, a post-Soviet state in Eastern Europe (*1*,*2*), and has been described sporadically in the neighboring provinces in northwestern and central Russia (*2*–*5*) and in Latvia (*6*). More important, it is multidrug resistant (MDR) (and most likely extensively drug resistant). In a recent Belarus study, SIT266 was found in 25 of 163 strains; all 25 were MDR (*1*). This situation contrasts clearly with its apparently parental type SIT264, which differs from SIT266 in a single spacer 8 (Figure, panel A). SIT264 is more widespread across Eastern Europe but at very low prevalence and is not associated with multidrug resistance (*3*,*6*,*7*). On the basis of 24 mycobacterial interspersed repetitive unit variable number tandem repeats clustering and robust phylogenetic single-nucleotide polymorphisms, SIT264 and SIT266 isolates are assigned to the Latin American–Mediterranean lineage of *M. tuberculosis* (*2*).

**Figure:**
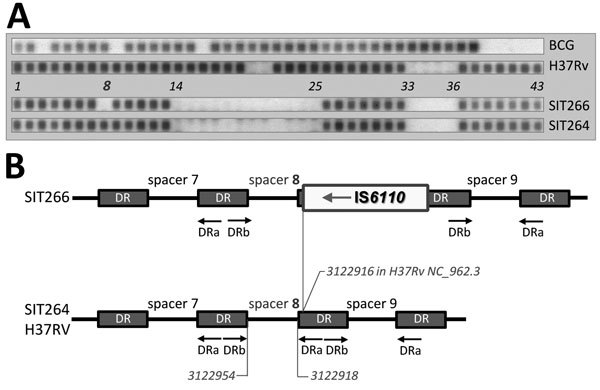
A) Spoligotyping hybridization profiles of H37Rv and BCG reference strains, *Mycobacterium tuberculosis* SIT266 (2 strains in this study) and SIT264 (previously published strain in [*3*]). SIT, spoligotype international type, designated according to SITVIT_WEB database (http://www.pasteur-guadeloupe.fr:8081/SITVIT_ONLINE). B) Schematic view of the DR/CRISPR locus (region of spacers 7–9) in spoligotypes SIT264 and SIT266 and reference strain H37Rv, inferred from next-generation sequencing data. Reverse DRa primer is biotin-labeled. IS*6110* is not to scale.

We recovered 2 MDR *M. tuberculosis* isolates of SIT266 from pulmonary tuberculosis patients from northwestern Russia in 2014. Bacterial DNA was subjected to macroarray-based spoligotyping (*8*) and whole-genome sequencing on the MiSeq platform (Illumina, San Diego, CA, USA). *M. tuberculosis* NGS data were deposited in the National Center for Biotechnology Information Sequence Read Archive (project no. PRJNA305488).

The short sequencing reads were subjected to analysis by using the SpoTyping program (https://github.com/xiaeryu/SpoTyping) (*9*) and the TGS-TB online tool (https://gph.niid.go.jp/tgs-tb/) (*10*) to deduce their spoligoprofile. We used the TGS-TB tool to map the IS*6110* insertion sites and detect drug resistance mutations. The reads also were mapped to the genome of reference strain H37Rv (GenBank accession no. NC_00962.3) by using the Geneious 9.0 package (Biomatters Ltd, Auckland, New Zealand). We obtained 1,294,895 and 816,693 paired reads for strains 4542 and 8279, respectively, and mapped them to the reference. Mean read length was 300 bp, and the average genome coverage was 72.

Strains 4542 and 8279 were phenotypically MDR and harbored mutations associated with resistance to all 5 first line-drugs. The macroarray hybridization spoligotyping assigned both strains to spoligotype SIT266. However, by in silico typing, their spoligotype was predicted to be SIT264 (Figure, panel A). To reconcile these findings, we hypothesized that this discrepancy resulted from an IS*6110* asymmetrically inserted in the direct repeat unit adjacent to the spacer 8 in a SIT266 isolate. This insertion would disrupt a target sequence for biotin-labeled DRa primer, thus preventing spacer 8 from amplification. Indeed, in both SIT266 isolates, the in silico analysis identified a forward IS*6110* insertion that was mapped to position 3122916 in H37Rv genome (Technical Appendix Figure). This location correlates with the location of spacer 8 in this same genome from positions 3122954 to 3122918. Thus, IS*6110* precedes spacer 8 in the genome of isolate with spoligotype SIT266, or follows it, within the DR locus (Figure, panel B; Technical Appendix Figure).

An immediate excellent contribution of NGS with regard to tuberculosis treatment and control is its capacity to rapidly screen for multiple gene targets linked to the development of drug resistance. However, knowledge of strain genotype is no less clinically and epidemiologically relevant. A superspreading strain might be marked with other pathobiologically important features. In the case presented here (indeed emerging and MDR), the NGS-based in silico spoligotyping would confuse the MDR/extensively drug resistant SIT266 with “less dangerous” SIT264. To be precise, the revealed discrepancy is not inherent to the NGS technology itself. Although the general limitation of the use of short sequencing reads to infer repetitive genome regions is known, it did not pose a problem in our study. However, both bioinformatics tools predicted the spoligoprofile solely from the presence or absence of spacer sequences and did not take into account a “hiding” effect exerted by a putative IS*6110* insertion on adjacent spacer under classical spoligotyping.

In conclusion, we suggest that an accurate NGS-based prediction requires an integrative approach to all relevant information obtained by in silico analysis of a given genome locus. In particular, not only presence of CRISPR spacers but also presence and location of potentially interfering IS*6110* insertion(s) should be considered for correct NGS-based assignment to internationally recognized spoligotypes.

Technical Appendix Whole-genome, short-sequencing reads of *Mycobacterium tuberculosis* strain 4542 mapped to complete genome of reference strain H37Rv (spacers 7–9 in CRISPR locus).

## References

[1] Zalutskaya A, Wijkander M, Jureen P, Skrahina A, Hoffner S. Multidrug-resistant *Myobacterium tuberculosis* caused by the Beijing genotype and a specific T1 genotype clone (SIT No. 266) is widely transmitted in Minsk. Int J Mycobacteriol. 2013;2:194–8. 10.1016/j.ijmyco.2013.08.001 26786121

[2] Mokrousov I, Vyazovaya A, Narvskaya O. *Mycobacterium tuberculosis* Latin American-Mediterranean family and its sublineages in the light of robust evolutionary markers. J Bacteriol. 2014;196:1833–41. 10.1128/JB.01485-13 24584500 PMC4011003

[3] Narvskaya O, Mokrousov I, Otten T, Vishnevsky B. Molecular markers: application for studies of *Mycobacterium tuberculosis* population in Russia. In: Read MM, editor, Trends in DNA fingerprinting research. New York: Nova Science Publishers; 2005. p. 111–25.

[4] Ignatova A, Dubiley S, Stepanshina V, Shemyakin I. Predominance of multi-drug-resistant LAM and Beijing family strains among *Mycobacterium tuberculosis* isolates recovered from prison inmates in Tula Region, Russia. J Med Microbiol. 2006;55:1413–8. 10.1099/jmm.0.46575-0 17005791

[5] Mokrousov I, Vyazovaya A, Solovieva N, Sunchalina T, Markelov Y, Chernyaeva E, Trends in molecular epidemiology of drug-resistant tuberculosis in Republic of Karelia, Russian Federation. BMC Microbiol. 2015;15:279. 10.1186/s12866-015-0613-3 26679959 PMC4683759

[6] Tracevska T, Jansone I, Baumanis V, Marga O, Lillebaek T. Prevalence of Beijing genotype in Latvian multidrug-resistant *Mycobacterium tuberculosis* isolates. Int J Tuberc Lung Dis. 2003;7:1097–103 . 14598971

[7] Pardini M, Niemann S, Varaine F, Iona E, Meacci F, Orrù G, Characteristics of drug-resistant tuberculosis in Abkhazia (Georgia), a high-prevalence area in Eastern Europe. Tuberculosis (Edinb). 2009;89:317–24 . 10.1016/j.tube.2009.04.002 19539531

[8] Mokrousov I, Rastogi N. Spacer-based macroarrays for CRISPR genotyping. Methods Mol Biol. 2015;1311:111–31. 10.1007/978-1-4939-2687-9_7 25981469

[9] Xia E, Teo YY, Ong RT. SpoTyping: fast and accurate in silico *Mycobacterium* spoligotyping from sequence reads. Genome Med. 2016;8:19. 10.1186/s13073-016-0270-7 PMC4756441 26883915

[10] Sekizuka T, Yamashita A, Murase Y, Iwamoto T, Mitarai S, Kato S, TGS-TB: total genotyping solution for *Mycobacterium tuberculosis* using short-read whole-genome sequencing. PLoS ONE. 2015;10:e0142951. 26565975 10.1371/journal.pone.0142951 PMC4643978

