# Supplementary material for: Next-Generation Sequencing of Mycobacterium tuberculosis
Source: Emerg Infect Dis. 2016 Jun;22(6):1127–9. doi: 10.3201/eid2206.152051 (PMC4880099; doi:10.3201/eid2206.152051)
Supplement: Technical Appendix — Whole-genome, short-sequencing reads of Mycobacterium tuberculosis strain 4542 mapped to complete genome of reference strain H37Rv (spacers 7–9 in CRISPR locus). [file 15-2051-Techapp-s1.pdf]

Article DOI: http://dx.doi.org/10.3201/eid2206.152051

Next-Generation Sequencing of *Mycobacterium tuberculosis*

Technical Appendix

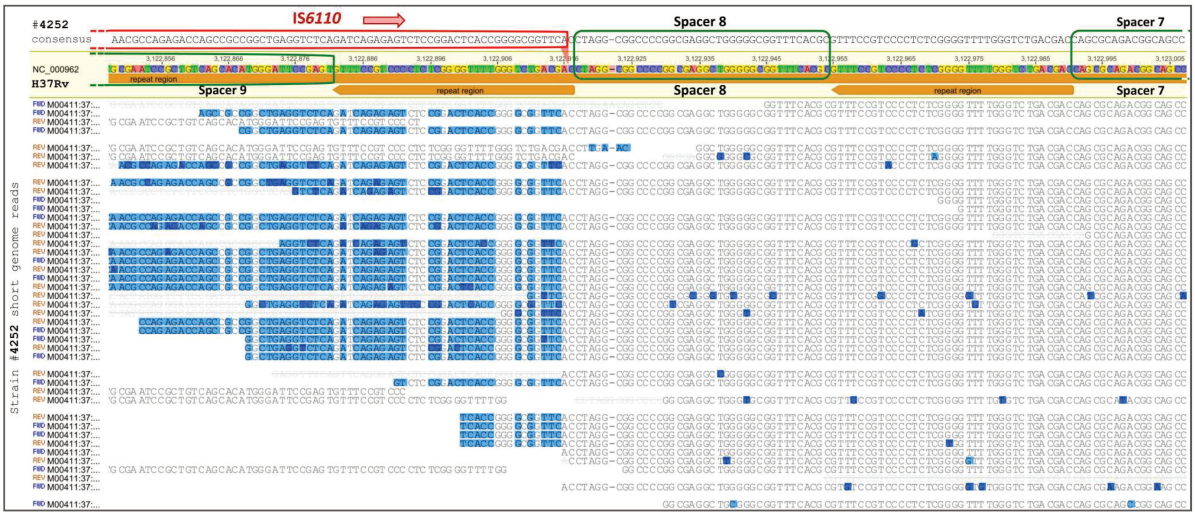

Technical Appendix Figure. Whole-genome, short-sequencing reads of *Mycobacterium tuberculosis* strain 4542 mapped to complete genome of reference strain H37Rv (NC_00962.3) by using Geneious 9.0 package: genome locus of spacers 7–9 in DR/CRISPR locus. Spacer numbers are given according to 43-spoligotyping (*1*). DR/CRISPR locus in *M. tuberculosis* is located on complementary strand, whereas spacers are consecutively numbered within the locus, not genome. Such a situation (IS*6110* asymmetrically inserted in the direct repeat unit immediately adjacent to a spacer) was discovered by Filliol et al. (*2*).

References

1. Kamerbeek J, Schouls L, Kolk A, van Agterveld M, van Soolingen D, Kuijper S, et al. Simultaneous detection and strain differentiation of *Mycobacterium tuberculosis* for diagnosis and epidemiology. J Clin Microbiol. 1997;35:907–14. PubMed

2. Filliol I, Sola C, Rastogi N. Detection of a previously unamplified spacer within the DR locus of Mycobacterium tuberculosis: epidemiological implications. J Clin Microbiol. 2000;38:1231–4. PubMed
